# FAMILY CONFLICT: A WELL KEPT SECRET IN LATINO DEMENTIA CAREGIVING

**DOI:** 10.1093/geroni/igz038.3228

**Published:** 2019-11-08

**Authors:** Gabriela Zaragoza, Lyda C Arevalo-Flechas, Bertha E Flores, Janna Lesser

**Affiliations:** 1 The University of Texas Health Science Center San Antonio, San Antonio, Texas, United States; 2 South Texas Veterans Heath Care System - Geriatrics and Extended Care Service, San Antonio, United States; 3 UT Health San Antonio, San Antonio, Texas, United States; 4 UT Health San Antonio School Of Nursing, San Antonio, Texas, United States

## Abstract

The number of Latino older adults living with Alzheimer’s disease and related dementias (ADRD) could increase more than six-fold, from fewer than 200,000 in 2004 to more than 1.3 million by 2050 . One-third of Hispanic households report having at least one family caregiver (36%). 63% of Latino family caregivers are in high burden situations compared to 51% of non-Latino caregivers. Although in recent years research with Latino caregivers has increased, studies examining the family as a unit in a culture in which familismo is a central value, remain limited. The purpose of this study was to describe the experience of Latino families caring for loved ones living with ADRD along the illness trajectory. Group interviews were conducted with families caring for individuals in different stages of dementia. Interviews were conducted in English or Spanish, transcribed verbatim, and analyzed in the source language by bilingual investigators. In contrast to the numerous positive aspects of caregiving reported by individual Latino caregivers in previous studies, preliminary analysis of family group interviews revealed predominantly negative perceptions of the family caregiving experience. Families reported conflict between family members, feelings of resentment, lack of family member involvement, and uneven distribution of caregiving duties. The complex nature of fulfilling family duties as dictated by familismo appears to have idealistic and realistic consequences for families. Further exploration of Latino family dynamics in the context of dementia caregiving is needed.

